# Interplay of Technology and Application of Gerontologic Biostatistics and Data Science for Career in Aging

**DOI:** 10.1093/geroni/igaf122.1380

**Published:** 2025-12-31

**Authors:** Michelle Shardell, Joanna Lynn Borgogna, Fatemeh Adelnia, Chixiang Chen, Shuo Chen, Jennifer Schrack

**Affiliations:** University of Maryland School of Medicine, Baltimore, Maryland, United States; Montana State University, Bozeman, Montana, United States; Vanderbilt University Medical Center, Nashville, Tennessee, United States; University of Maryland School of Medicine, Baltimore, Maryland, United States; University of Maryland School of Medicine, Baltimore, Maryland, United States; Johns Hopkins Bloomberg School of Public Health, Baltimore, Maryland, United States

## Abstract

The term ‘gerontologic biostatistics’ was introduced in 2010 and recently updated to include data science to describe a collection of analytical methods that addresses the challenging features of research data in studies of older adults. Many of the advances in gerontologic biostatistics and data science (GBDS) that have occurred in the past ∼15 years were motivated by new data modalities generated from recently developed technologies. As a result, the ability of early career scientists to leverage these data for innovative research depends on their understanding of the underlying technologies that produced these data. Herein, we will describe three such technologies, the data they generate, and examples of studies conducted by analyzing these data using GBDS methods. Specifically, we describe 16S rRNA amplicon sequencing technology that characterizes relative abundance of microbial taxa, accelerometers that provide objective measurements of physical activity, and magnetic resonance imaging-based multimodal neuroimaging that measures both brain structure and function. Specific examples include longitudinal cervicovaginal microbiota and reproductive aging among 750 women enrolled in the Human Papillomavirus in Perimenopause study, the role of physical activity in maintaining muscle oxidative capacity among 384 adults enrolled in the Baltimore Longitudinal Study of Aging, and age-related differences in whole-brain connectome networks among 39,675 adults enrolled in UK Biobank and 499 adults enrolled in the Lifespan Human Connectome Project in Aging study. These research studies exemplify advantages for scientists—at any career stage—to understand technological developments, the data they generate, and the application of GBDS for aging research.

